# Translating current biomedical therapies for long duration, deep space missions

**DOI:** 10.1093/pcmedi/pbz022

**Published:** 2019-11-15

**Authors:** Sonia Iosim, Matthew MacKay, Craig Westover, Christopher E Mason

**Affiliations:** 1 Department of Physiology and Biophysics, Weill Cornell Medicine, New York, NY 10021, USA; 2 The HRH Prince Alwaleed Bin Talal Bin Abdulaziz Alsaud Institute for Computational Biomedicine, Weill Cornell Medicine, New York, NY 10021, USA; 3 The Feil Family Brain and Mind Research Institute, Weill Cornell Medicine, New York, NY 10021, USA; 4 The WorldQuant Initiative for Quantitative Prediction, Weill Cornell Medicine, New York, NY 10021, USA

**Keywords:** NASA, spaceflight, International Space Station, NASA Twin Study, multi-scale omics

## Abstract

It is been shown that spaceflight-induced molecular, cellular, and physiologic changes cause alterations across many modalities of the human body, including cardiovascular, musculoskeletal, hematological, immunological, ocular, and neurological systems. The Twin Study, a multi-year, multi-omic study of human response to spaceflight, provided detailed and comprehensive molecular and cellular maps of the human response to radiation, microgravity, isolation, and stress. These rich data identified epigenetic, gene expression, inflammatory, and metabolic responses to spaceflight, facilitating a better biomedical roadmap of features that should be monitored and safe-guarded in upcoming missions. Further, by exploring new developments in pre-clinical models and clinical trials, we can begin to design potential cellular interventions for exploration-class missions to Mars and potentially farther. This paper will discuss the overall risks astronauts face during spaceflight, what is currently known about human response to these risks, what pharmaceutical interventions exist for use in space, and which tools of precision medicine and cellular engineering could be applied to aerospace and astronaut medicine.

## Introduction

Previous studies have demonstrated numerous human spaceflight-induced complications, such as cardiovascular alterations, bone and muscle loss, ocular dysfunction, risk of malignancy, hematological issues, and behavioral changes.^1–9^ In the past decade, omics studies have given us a closer look at cellular processes that indicate genetic, transcriptional, translational, inflammatory, and metabolic responses to the space environment. More recently, the NASA Twin Study^10^ created a unique, multi-omics analysis of a pair of monozygotic twins that examined spaceflight-related changes on a more comprehensive level than has ever been done before. This study used recent technological advances to create extensive molecular maps of the cellular and genetic changes that occur in astronauts, including alterations in DNA, RNA, proteins, lipids, metabolites, and the microbiome. This has also given us new insight into how radiation, microgravity, isolation, and stress affect the body, as well as new tools for monitoring changes in astronaut health.

Building on this work, many space agencies and research groups are now focusing on leveraging the methods of “precision medicine” to increase the safety of astronaut missions and improve long-term astronaut health and safety. Further advances have been made on Earth for cellular therapeutics, which are continuously changing the paradigm of treatment for many diseases—especially in cancer. By taking examples from regenerative medicine and immune-oncology, we can begin to design cellular therapeutics that may further protect astronauts and allow for missions of longer duration.

According to NASA, there are five main hazards of human spaceflight: radiation, gravity, distance (from Earth), hostile enclosed environments, and isolation. Although it may be challenging to dissociate these hazards from one another when studying an astronaut’s response to spaceflight, all hazards must be addressed and appropriately assessed before each mission. As an example, the distance from Earth (or a future-base) forces astronauts to be more self-reliant because of the increased time delay of communications as well as the finite supply of non-replenishable goods. Further, both isolation and hostile environments play major roles in the success of a mission and must be carefully planned for with specific astronauts in mind.

In this paper, we discuss the responses of the human body to spaceflight, with an emphasis on specific changes caused by radiation and micro gravity. We discuss new findings from the Twin Study, including potential risks for future astronauts during longer, or farther, missions from Earth. We cover pharmaceuticals in space as well as current therapies, clinical trials, and medical paradigms on Earth, which may be the building blocks for future astronaut medicine. This includes translation of cellular therapies, genetically engineered therapies, next-generation sequencing, multi-omic analyses, customized antibiotics, and precise nutritional regimes based on an astronaut’s microbiome.

## Spaceflight hazard: radiation

**Figure 1:**
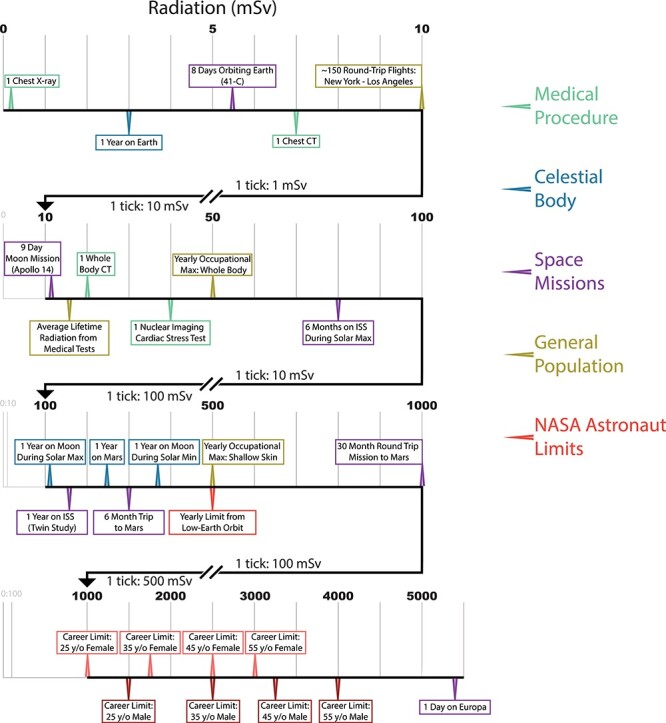
Relative radiation exposure (varying durations): medical procedures (green), the impact while on various celestial bodies (blue), specific space missions (purple), general population facts (gold), and recommended astronaut limits (red).

Radiation is a clear spaceflight risk, particularly the strong galactic cosmic rays (GCRs) that are mostly protected against by the Earth’s atmosphere and magnetosphere. Radiation damages DNA, RNA, proteins, and lipids, and increases overall oxidative stress within cells. Although the health effects of acute radiation exposure have been comprehensively studied, much less is known about the effects of chronic exposure for astronauts undertaking long missions outside Earth’s orbit. Also, it is important to note that being exposed to a large dose of radiation within a short time-frame will be more dangerous than the same dosage over a longer duration, primarily because of cellular turnover. To put radiation exposure into the context of spaceflight, we aggregated multiple sources to visualize relative radiation exposure from medical tests, time on a celestial body within our solar system, the duration of select missions, and finally the current career-long occupational maximum radiation exposure for astronauts (Fig. 1).

Each type of exposure can carry very different degrees of risk. As an example, a year on Earth will expose an average human to 3 milliSieverts (3 mSv), whereas the maximum whole-body occupational exposure for a year on a job for radiation workers in the United States is 50 mSv. Notably, 1 year on the surface of the Moon would likely expose an astronaut to 100–350 mSv, depending on solar conditions. When traveling to other celestial bodies, radiation will be a primary risk factor. Some celestial bodies, such as Titan, would expose astronauts to lethal limits within a day (>5,000 mSv), whereas other bodies would expose astronauts to less radiation than that to which humans are normally exposed (e.g., Venus). To put this in the context of an astronaut, one 30-month round trip mission to Mars (~6-month trips each way with 18 months on the surface) would put a 25-year-old female at her career limit, whereas this same person could be on the Moon (approximately the same yearly radiation as Mars) for roughly 4 years. Simply put, the transit time is so much less to get to the Moon, that the risk is decreased. Further, a male, 55-year-old astronaut could have accomplished either of these missions four times before meeting his career limit.

The main concerns with radiation are pathological vascular changes, genetic mutations, immune dysfunction, and cancer.^4^^,^^5^^,^^10^ Epidemiological studies have suggested a latent relationship between radiation exposure and cardiovascular disease,^6–9^ as well as with clonal hematopoiesis,^11^ and physiologic studies in mice have shown long-lasting changes in cardiomyocyte gene expression and cellular signaling. The astronaut career limits are set based on a permissible career exposure limit of a 3% increased risk of exposure-induced death from cancer, estimated at a 95% confidence interval using a system developed by the National Council on Radiation Protection and Measurements that accounts for individual differences in risk (e.g., age, gender, smoking history).^3^ However, a study of former astronauts found no significant correlation between space radiation dose and cardiovascular or cancer-associated mortality.^2^ The study recognizes its small sample size and statistical limitations, but it points out that much remains to be learned about long-term outcomes of space radiation exposure and the actual extent of the threat that it poses.

Nonetheless, leukemias are known to be among the most common radiation-induced cancers and pose a particular risk because they can develop relatively rapidly.^12^ A study showed that simulated solar energetic particle and GCR radiation led to DNA damage and altered differential potential of human hematopoietic stem cells, indicating a risk of malignancy and signs of immune dysfunction.^13^ Furthermore, it showed a distinct difference in gene expression not only in irradiated versus control cells, but also a difference between cells exposed to different types of radiation. Numerous other studies found changes in regulation of the immune system, altered interactions between innate and adaptive components of the immune system,^14^ and significant dampening of lymphocyte response to stimuli.^15^ These features may also alter the risk of clonal hematopoiesis and the emergence of mutated alleles.^16^^,^^17^ Additionally, it was found that latent herpes virus was reactivated in subjects during a 6-month spaceflight.^14^

## Spaceflight hazard: gravity

There have been many studies on the health effects from microgravity, most significantly effects on the cardiovascular and musculoskeletal systems. It is known that a headward shift of fluid occurs, causing maladaptive changes that put stress on blood vessels and the heart. Furthermore, other studies have shown that the resulting increased cranial pressure may induce optic disc edema that can lead to choroidal folds, a pathology that has been termed “spaceflight-associated neuro-ocular syndrome”, or SANS. The decreased gravitational load also leads to muscle atrophy and bone loss. In the years leading up to the Twin Study, studies focused on the molecular changes that can occur because of microgravity. Researchers have been aiming to uncover pathologic mechanisms and unanticipated effects using off-target profiling. For instance, microgravity and simulated microgravity experiments have shown elevations in myostatin (a protein that inhibits muscle growth),^18^ decrease in PGC-1α (peroxisome proliferator-activated receptor gamma coactivator-1 alpha), a regulator of mitochondrial proliferation,^19^^,^^20^ and increased physiological and cognitive sensitivity to carbon dioxide levels. Transcriptome studies found upregulation of the antibacterial response protein classes of actin and actin-related proteins, and of oxytocin- and serotonin-mediated pathways.

Microgravity simulations have shown that plasma levels of both coagulative and fibrinolytic proteins decrease, impacting the blood-clotting cascade, and they showed that a protein hormone biomarker of cardiovascular workload (NT pro-BNP) increases.^21^ Furthermore, these kinds of simulations have shown that microgravity induces cytoskeletal alterations that affect a whole range of cellular processes, including proliferation, migration, and signal transduction.^22^ Renal proteins serving as biomarkers of electrolyte regulation were altered, likely because of the fluid shifts that occur, overall decrease in plasma volume, and increased intake of salt additives. One study examining astronaut urinary proteome found that three proteins, glucosidase alpha acid (GAA), heparan sulfate proteoglycan (HSPG2), and alanyl aminopeptidase (ANPEP), do not return to baseline levels post-spaceflight, possibly correlating to changes in cytoskeletal reorganization, angiogenesis, extracellular matrix reorganization, and some features of hormone metabolism.^5^ Additional proteomic changes that have been detected include increased production of cytokines (and cortisol),^22^ changes in regulators of aerobic metabolism, and decrease in muscle and bone protein metabolism.^18^^,^^23^

## Human response to a year in space

Previous work has been done to analyze how multifactorial hazards of spaceflight affect human functions, such as psychomotor and cognitive processing. Declines in movement speed, movement accuracy, internal timekeeping, and limb position sense have been identified. Changes in vestibular orientation cause “space motion sickness” in up to 70% of astronauts during the first week in orbit. There has also been evidence of difficulty with prefrontal functions, including decision-making, attention, concentration, and spatial working memory. Additionally, issues with mood, depression, anxiety, and irritability have been documented. Overall, there appears to be a negative impact on neurocognitive performance,^24^ likely because of a combination of psychological stress, change in sleep, physiological stress, and perhaps even the effects of radiation, although the majority of these studies were relatively short term.

Within the Twin Study, two identical twins were examined over 5 months before flight, 1 year when one twin was on the International Space Station (ISS), and another eight months post-flight. This study is the first of its kind to examine the effects of space at the molecular, cellular, physiological, and cognitive levels while tracking an identical, Earth-bound, twin at the same time. The Twin Study analyzed 18 different sample types (including different blood cell fractions) and 20 different analyses (ranging from cognitive tests to cytokine profiling or RNA-sequencing) across over 300 samples (including cell type fractions) (Table 1).^10^

**Table 1:** Sample types and analyses conducted by flight time during the NASA twin study.

Flight time	Sample type	Analysis
Pre-flight	Blood (plasma)	Biochemistry
In-flight	Blood (plasma)	Biochemistry
Post-flight	Blood (plasma)	Biochemistry
Pre-flight	Blood (plasma)	Cytokine profiling
In-flight	Blood (plasma)	Cytokine profiling
Post-flight	Blood (plasma)	Cytokine profiling
In-flight	Blood (plasma)	Oxidative status
Post-flight	Blood (plasma)	Oxidative status
Pre-flight	Blood (plasma)	Oxidative stress and inflammation
In-flight	Blood (plasma)	Oxidative stress and inflammation
Post-flight	Blood (plasma)	Oxidative stress and inflammation
Pre-flight	Blood (PBMCs)	qRT-PCR (T:A)
In-flight	Blood (PBMCs)	qRT-PCR (T:A)
Post-flight	Blood (PBMCs)	qRT-PCR (T:A)
Pre-flight	Blood (PBMCs)	qRT-PCR TRAP
In-flight	Blood (PBMCs)	qRT-PCR TRAP
Post-flight	Blood (PBMCs)	qRT-PCR TRAP
Pre-flight	Blood (CD19)	RNA-seq
Pre-flight	Blood (CD4)	RNA-seq
Pre-flight	Blood (CD8)	RNA-seq
Pre-flight	Blood (LD)	RNA-seq
Pre-flight	Blood (PBMCs)	RNA-seq
In-flight	Blood (CD19)	RNA-seq
In-flight	Blood (CD4)	RNA-seq
In-flight	Blood (CD8)	RNA-seq
In-flight	Blood (PBMCs)	RNA-seq
In-flight	Blood (LD)	RNA-seq
Post-flight	Blood (CD19)	RNA-seq
Post-flight	Blood (CD4)	RNA-seq
Post-flight	Blood (CD8)	RNA-seq
Post-flight	Blood (LD)	RNA-seq
Post-flight	Blood (PBMCs)	RNA-seq
Pre-flight	Blood (plasma)	Targeted metabolomics
In-flight	Blood (plasma)	Targeted metabolomics
Post-flight	Blood (plasma)	Targeted metabolomics
Pre-flight	Blood (CD4)	TCR
Pre-flight	Blood (CD8)	TCR
Pre-flight	Blood (PBMCs)	TCR
In-flight	Blood (CD4)	TCR
In-flight	Blood (CD8)	TCR
In-flight	Blood (PBMCs)	TCR
Post-flight	Blood (CD4)	TCR
Post-flight	Blood (CD8)	TCR
Post-flight	Blood (PBMCs)	TCR
Pre-flight	Blood (T-cells)	Telo-FISH/dGH
In-flight	Blood (T-cells)	Telo-FISH/dGH
Post-flight	Blood (T-cells)	Telo-FISH/dGH
Pre-flight	Blood (plasma)	Untargeted metabolomics
In-flight	Blood (plasma)	Untargeted metabolomics
Post-flight	Blood (plasma)	Untargeted metabolomics
Pre-flight	Blood (plasma)	Untargeted proteomics
In-flight	Blood (plasma)	Untargeted proteomics
Post-flight	Blood (plasma)	Untargeted proteomics
Pre-flight	Blood (CD4)	WGBS
Pre-flight	Blood (CD8)	WGBS
In-flight	Blood (CD4)	WGBS
In-flight	Blood (CD8)	WGBS
Post-flight	Blood (CD4)	WGBS
Post-flight	Blood (CD8)	WGBS
Pre-flight	Body	Body mass
In-Flight	Body	Body Mass
Post-Flight	Body	Body Mass
Pre-Flight	Body	Cardiac and Vascular
Ultrasound		
In-flight	Body	Cardiac and vascular ultrasound
Post-flight	Body	Cardiac and vascular ultrasound
Pre-flight	Body	Vascular structure and function
In-flight	Body	Vascular structure and function
Post-flight	Body	Vascular structure and function
Pre-flight	Cognition	Cognition
In-flight	Cognition	Cognition
Post-flight	Cognition	Cognition
Pre-flight	Fecal	Metagenome
In-flight	Fecal	Metagenome
Post-flight	Fecal	Metagenome
Pre-flight	Ocular	Ocular imaging
In-flight	Ocular	Ocular imaging
Post-flight	Ocular	Ocular imaging
Pre-flight	Urine	Biochemistry
In-flight	Urine	Biochemistry
Post-flight	Urine	Biochemistry
Pre-flight	Urine	Oxidative stress and inflammation
In-flight	Urine	Oxidative stress and inflammation
Post-flight	Urine	Oxidative stress and inflammation
Pre-flight	Urine	Targeted metabolomics
In-flight	Urine	Targeted metabolomics
Post-flight	Urine	Targeted metabolomics
Pre-flight	Urine	Targeted proteomics
In-flight	Urine	Targeted proteomics
Post-flight	Urine	Targeted proteomics
Pre-flight	Urine	Untargeted proteomics
In-flight	Urine	Untargeted proteomics
Post-flight	Urine	Untargeted proteomics
Pre-flight	Blood (plasma)	Oxidative status
Pre-flight	Blood (CD4)	qRT-PCR (T:A)
Pre-flight	Blood (CD8)	qRT-PCR (T:A)
Pre-flight	Blood (LD)	qRT-PCR (T:A)
Pre-flight	Blood (CD19)	qRT-PCR (T:A)
Post-flight	Blood (CD19)	qRT-PCR (T:A)
Post-flight	Blood (CD4)	qRT-PCR (T:A)
Post-flight	Blood (CD8)	qRT-PCR (T:A)
Post-flight	Blood (LD)	qRT-PCR (T:A)
In-flight	Blood (ambient return) (CD19)	qRT-PCR (T:A)
In-flight	Blood (ambient return) (CD4)	qRT-PCR (T:A)
In-flight	Blood (ambient return) (LD)	qRT-PCR (T:A)
In-flight	Blood (ambient return) (PBMCs)	qRT-PCR (T:A)
In-flight	Blood (ambient return) (CD8)	qRT-PCR (T:A)
In-flight	Blood (ambient return) (PBMCs)	qRT-PCR TRAP
In-flight	Blood (ambient return) (CD4)	RNA-seq
In-flight	Blood (ambient return) (CD8)	RNA-seq
In-flight	Blood (ambient return) (LD)	RNA-seq
In-flight	Blood (ambient return) (T-cells)	Telo-FISH/dGH
In-flight	Blood (ambient return) (CD4)	WGBS
In-flight	Blood (ambient return) (CD8)	WGBS

Flight time references when samples were collected compared to time on ISS. Sample type abbreviations: lymphocyte depleted (LD) cells and peripheral blood mononuclear cells (PBMCs). Analysis abbreviations: RNA-sequencing (RNA-seq), T-cell receptor sequencing (TCR), telomere fluorescence in situ hybridization (Telo-FISH), quantitative real-time polymerase chain reaction (qRT-PCR), whole-genome bisulfite sequencing (WGBS).

There were several key findings suggestive of significant stress on the body, many confirming previous findings, and some that gave us new insight. Strikingly, telomere lengthening occurred, but the mechanism and the health consequences of this finding are not currently known. However, telomeres rapidly shortened upon return to Earth, and the amount of critically short telomeres actually increased after return to Earth. Telomere dysregulation may play a role in cell aging, cell death, and may be a determinant of cancer risk in astronauts. Chromosome translocations increased both during and after spaceflight, further contributing to risk of malignancy and indicating an ongoing process to repair the genome after spaceflight. Locus-specific epigenetic changes and transcriptional alterations showed enrichment in DNA repair pathways, further supporting this result.

Significant shifts also occurred in immune function pathways, indicating the presence of immune stress and inflammation. There has been concern about dampening of immune function, but a vaccination response experiment showed that T-cell response was adequately mounted (as measured by T-cell receptor repertoire, or TCR, diversity). Metabolic changes were also observed, including altered amino acid metabolism, increased pro-inflammatory lipids, increased lactic acid production, and decreased mitochondrial respiration. Microbiome alterations were noted, both in species richness and microbe functionality, but species diversity was not significantly affected. The implications of these changes are not yet known, but are unlikely to be severe.

Furthermore, proteomic data aligned with the well-known physiological changes that occur in the space environment. Changes occurred in fluid and blood pressure regulation proteins, vascular remodeling proteins, and insulin-binding proteins that play a role in body mass and muscular deconditioning. Signs of cardiovascular inflammation and carotid artery thickening were found, and it is not currently known if this alteration is reversible. There was also evidence of SANS, which was expected given results from previous astronauts.

As in previous studies, cognitive measures showed a general decrease in speed and accuracy, but with a couple of nuanced findings. Cognitive speed actually initially increased during early in-flight testing—the decline in function occurred most significantly during the 6-month post-flight period. Previous studies have indicated that stress and radiation may decrease synaptic density within the brain, and that radioactive nucleotides may cross the blood-brain barrier and play a role in decline. Additional work needs to be done to better understand the mechanisms behind the cognitive decline, and to clarify how exactly microgravity, physiological and psychological stress, and radiation target the brain.

An important insight gained from the Twin Study is a better recognition of temporality of changes that occur during long-term exposure to space conditions. Levels of risk and types of dysfunction change are based on the duration of the exposure. There seems to be a distinguishable difference between the differentially expressed genes during the first 6 months of the trip compared with the last 3 months of the trip. Significantly greater transcriptional changes occurred during the second half of the mission, starting at month 6. Also, the number of genes that were differentially expressed and regulated increased 7-fold in the last 6 months of the mission.

While many of these changes returned to baseline upon return to Earth, a subset of genes had altered expression that persisted into the 6-month post-flight period. This raised some concern that spaceflight may permanently alter the expression of several genes, but it is too early to know from just this study alone. Inflammation signatures also showed an interesting temporal pattern. Cytokine levels were subdivided into three categories: those that increased above their baseline following spaceflight, those that decreased below their baseline following spaceflight, and those that showed a rapid and drastic increase upon return from spaceflight but then returned to baseline levels. This may indicate that some immune and inflammatory processes restabilized after space exposure, whereas others are upregulated or downregulated for an extended period of time.

There are a few high-risk categories that require special attention, in terms of monitoring and potential intervention. Astronauts appear to be particularly vulnerable to cardiac disease, musculoskeletal dysfunction, and ocular remodeling so it would be important to track cardiovascular function, fluid shifts, measures in musculoskeletal decline, and changes in vision. Cognitive function is another critical category that requires ongoing testing and evaluation, which consists of pre- and post-flight interviews and targeted examinations. This includes tests such as Visual Object Learning, Abstract Matching, Line Orientation, Emotion Recognition, etc.

## Pharmaceuticals in space

Mitigating the adverse effects on astronaut health has been difficult and mostly includes interventions such as exercise, nutrition, and some medications. According to a 2017 report from NASA, the ISS currently has a total of 107 medications included in the medical kits.^25^ The study of pharmacotherapy in space has been limited but is crucial to astronaut health for both current and future missions. The medications used during spaceflight have primarily been for symptomatic relief, such as sleeping difficulties, fatigue, motion sickness, gastrointestinal problems, congestion, but have also included anti-infectives, bisphosphonates (to mitigate bone loss), and vitamins.^26–32^ The changes that occur in blood flow, plasma and extravascular volumes, dehydration, vascular permeability, and slowed gastrointestinal transit time may alter drug absorption, distribution, and elimination.^33–35^ Additionally, increased homogeneity of pulmonary perfusion in space (resulting in greater diffusion capacity)^36^^,^^37^ and higher deposition of aerosols inhaled in microgravity^38^^,^^39^ may increase the effect of aerosolized medications and their toxicities (salmeterol and fluticasone are two types of inhalers available onboard the ISS).^40^ Alterations in pharmacokinetics from these fluid shifts and effects of microgravity have only been objectively evaluated in a few drugs. Acetaminophen has been determined to have some delay in absorption and scopolamine kinetics were indeterminate; thus far, no modifications have been made to the prescription of these drugs in space.^33^^,^^41^^,^^42^

Liver function is fundamental to processing many types of medications, and data suggest that hepatic enzyme expression changes during prolonged spaceflight. These findings are based on antipyrine metabolism, which is a biomarker for the function of several key cytochrome p450 isoenzymes (however, no phenotypic data have yet been produced).^43–45^ The mechanisms causing these changes are currently unknown but are postulated to be related to alterations in hormonal and cytokine profiles as well as changes in DNA methylation.^24^ Shifts in kidney function and cellular transport proteins are also critical to pharmacokinetics, but the significance of these changes to processing pharmaceuticals in space has yet to be determined. The assessment of pharmacodynamics in space has been minimal and largely inconclusive, so further studies need to be done to evaluate drug potency in space.^46^

Additional concerns include altered bacterial virulence and chemical instability and degradation of medications aboard spacecraft.^47–54^ Compounded with the alterations in immune function that occur in astronauts, failure of antibiotic agents could be catastrophic for crewmembers.^46^ Conversely, upregulated immune activity may cause hypersensitivity reactions to medications.^47^ Lastly, it is important to note that many common medications can prolong cardiac QTc intervals (which increases risk for sudden cardiac death), so the fact that some studies showed that long-duration spaceflight increased QTc times is also concerning.^55^

## Engineering therapeutics for human space exploration

As we gain more molecular data on human response to spaceflight, we will be able to further tailor specific therapies and identify necessary medications to limit potential complications during long duration, deep space missions where resupplying will not be an option. From previous work we already knew the effects of spaceflight on bone and muscle atrophy, ocular changes, and cardiac changes. From the Twin Study we now have a molecular level understanding of drastic inflammation and cytokine responses during flight and upon re-entry. As this type of study is expanded to a larger population, we will begin to identify additional changes within specific individuals and astronauts in general. It may also open up means of genetic engineering for cells, of which many more options exist today than ever before.

The USA launched the first genetically engineered clinical trial in 1989, and many other countries have since begun various genetically engineered trials.^56^ From this came the invention of chimeric antigen receptors (CARs), which may persist long-term within patients. The first two FDA-approved CAR T-cell (CAR-T) therapies (Axicabtagene Ciloleucel^57^ and Tisagenlecleucel^58^) were announced within the last few years. To create CAR-T therapies, T-cells are extracted from the patient or a donor, genetically engineered, expanded, and then infused into the patient to infiltrate any tissue system other T-cells can access to specifically target cells based on their single chain variable fragment.

Given the recent rise in genetic engineering and cellular therapeutic trials, future astronauts may be given cellular preventatives or therapies to improve response to spaceflight and help ameliorate known hazards. One such example would be to engineer cells to have superior radiation-resistance abilities. Thankfully, Earth is full of diverse life forms with highly specialized and unique abilities so we are not forced to completely re-invent the wheel when it comes to trying to improve human response to new environments. Given that the main negative effect from radiation is directed towards DNA damage, it may make sense that improved DNA protection allows for cellular survivability and escapes from radiation-induced health consequences.

As such, research has been conducted into these radiation-tolerant mechanisms. Dsup, a protein found in tardigrades (an organism known for its radio-resistant abilities compared to humans) which has been shown to enhance DNA protection, is one potential candidate to improve overall human radiation-resistance. As a proof of concept, it has been shown that expression of Dsup within human cell lines improves their overall radio-resistance when exposed to 1 Gy of irradiation.^59^

Beyond this, other animals exist with superior anti-tumor effects. As an example, consider elephants which are much larger than the average human, and so containing many more cells, and further are likely to be exposed to more UV radiation. However, even given these characteristics which may otherwise increase susceptibility to cancer, elephants have a lower incidence of cancer relative to humans. Research has identified one potential reason for the decreased risk of cancer in elephants—20 copies (10 times more than an average human) of the prominent tumor-suppressor gene TP53.^60^ Given the decrease incidence of cancer, it may make sense to increase the overall copy number of TP53 within human cells, as long as the expression is controlled and stable, especially if the person is expected to be exposed to a large amount of radiation.

Although these two examples would be best if applied to all cells within the body, a safer and likely more realist intervention would be to engineer the immune system directly. As an example, in a breakthrough concept paper in *Cell*, a group of researchers were able to identify a target which is ubiquitously expressed on differentiated myeloid cells, including cancerous acute myleoid leukemia (AML) cells, which is not essential for the development of these cell types, CD33. As a result, they engineered a “new” immune system through the genetic inactivation of this gene from hematopoietic stem cells (HSCs) which were then transfused into rhesus monkeys with AML.^61^ This resulted in the creation of a tumor-specific neo-antigen through the removal of the antigen on normal cells while it persists on cancer cells. CD33 is already targeted within CAR clinical trials; however, side effects are likely unpreventable without this type of normal-tissue engineering before treatment.^62^

Further engineering could be conducted to allow for immune-protected cells which are capable of secreting necessary molecules to improve or stabilize humans during spaceflight. For example, regenerative medicine for diabetes involves the differentiation of cells into islets using either donor cells, such as embryonic stem cells (ESCs), or patient cells, such as induced pluripotent stem cells (iPSCs).^63^ These therapies then act as if they were the patient’s pancreas, capable of producing insulin in response to glucose. These therapies can further engraft to the patients in such a way that they are immune-privileged, where the encapsulation device does not allow for immune cell infiltration, or have direct engraftment and engagement with the host immune system. Such a paradigm would allow for a singular treatment every few months, or potentially years, as opposed to daily insulin dependency. If proven successful, this methodology could theoretically be applied to safely implant any engineered cell to aid in immune response, metabolism, or secretion of specific molecules in response to a stimulus or constitutively.

Taken together, these unique adaptations along with others could be combined to improve overall human health, response to spaceflight, and even response to specific hazards of space. The first introduction of these ideas will likely be through engineering the hematopoietic system. As an example, an astronaut’s own HSCs can be immobilized through injection of Granulocyte-colony stimulation factor (G-CSF). These cells can further be engineered to express Dsup, incorporate multiple copies of TP53, and even introduce specific variants within EPAS1. Together this may allow for an overall increase in radio-resistance, decreased susceptibility to blood cancers, and decreased need for oxygen. These and other improvements should be applied to all tissue systems, or, ideally, specific tissue systems other than hematopoietic, as such specific modification would be less invasive and capable of high-quality control *in vitro* measurements before cells were re-infused. Of course, extensive pre-clinical models (mouse, primate) will need to be explored within these contexts to ensure their efficacy and safety. It may not be far-fetched to imagine a world where, once improved genetic engineering technologies have been developed, in vivo somatic engineering could also then take place.

Precision medicine could also be vital for measuring and maximizing efficacy of medications in astronauts. Minimally invasive tests, such as those utilizing liquid biopsies, would be fundamental for personalized omics studies regarding pharmaceutical agents. Expression of hepatic cytochrome p450 enzymes, cellular transport proteins, hormonal and cytokine profiles, DNA methylation analysis, and microbiome genomics and metabolomics can all be directly applied to tailoring effective medical treatment for humans in space.

Finally, microbiome therapies are also a key component of the astronaut’s health,^64^ the general environment,^65^ and also could be customized to ensure continued diversity and overall function. While ratios of the Firmicutes and Bacteroidetes changed in a potentially negative direction in the Twin Study, they did revert back to normal upon return to Earth. In future studies, probiotics could be administered to prevent this shift, and continuous monitoring of the microbiome of the astronauts could optimize this approach. Notably, it has already been shown that DNA sequencing can reliably function in microgravity,^66^ enabling sequencing on the ISS^67^ as well as detection of modified nucleic acids^68^ in the microbes on the ISS, meaning the technology is also in place for continual precision metagenomics^69^ for the astronauts.

## Conclusions

The effects of spaceflight on the human body extend from physiological changes at the level of tissue structure and fluid compartments, down to molecular alterations in DNA, RNA, and protein. The NASA Twin Study has given us a detailed look at the omics of spaceflight and shed light on some of the most important shifts that can occur, yet there is still much left to learn. Additional studies will be required to extrapolate the data, develop cutoffs, create quantitative guidelines, and shape algorithms for flight surgeons to monitor and intervene when necessary. In the near future we will likely begin to see new cellular therapy paradigms which exist in the clinic to be applied to astronauts to help combat the hazards of spaceflight and further missions away from Earth. Furthermore, new assays and metrics of health and disease will invariably emerge that we cannot predict today. Thus, having viably frozen cells and fluids will be essential to future-proof the collection protocols as much as possible. Moreover, the expansive, recent work on cell-free DNA and RNA metrics that can serve as a “molecular whole body scan” also show great promise, indicating that care in storing and preserving all samples will be beneficial to future astronauts in space as well as patients here on Earth.

Precision medicine will be vital for measuring and maximizing efficacy of medications in astronauts. Expression of hepatic cytochrome p450 enzymes, cellular transport proteins, hormonal and cytokine profiles, DNA methylation analysis, and microbiome genomics and metabolomics can all be directly applied to tailoring effective medical treatment for humans in space. Further, sequencing in space, as shown with Oxford Nanopore, can empower astronauts with immediate and actionable data in regard to their own health or experiments being conducted. Further preventative treatments and enhancements may be possible in the near future through the use of cellular engineering therapeutics. As more genetically engineered therapies are approved by the FDA and more long-term studies have been completed, we will likely begin to apply these types of paradigms to address newly found spaceflight hazards and complications.
